# Hypoglycemia in blood glucose level in type 2 diabetic Japanese patients by continuous glucose monitoring

**DOI:** 10.1186/s13098-019-0412-3

**Published:** 2019-02-15

**Authors:** Maiko Hajime, Yosuke Okada, Hiroko Mori, Fumi Uemura, Satomi Sonoda, Kenichi Tanaka, Akira Kurozumi, Manabu Narisawa, Keiichi Torimoto, Yoshiya Tanaka

**Affiliations:** First Department of Internal Medicine, School of Medicine, University of Occupational and Environmental Health Japan, Kitakyushu, 807-8555 Japan

**Keywords:** Hypoglycemia, Type 2 diabetes mellitus, Continuous glucose monitoring

## Abstract

**Background:**

Hypoglycemia is associated with cardiovascular diseases, increased risk of death. Therefore, it is important to avoid hypoglycemia. The aim of this study was to characterize hypoglycemia according to glycated hemoglobin (HbA1c) level and determine the contributing factors in type 2 diabetes mellitus (T2DM), using continuous glucose monitoring (CGM).

**Methods:**

T2DM patients (n = 293) receiving inpatient care were divided into five groups according to HbA1c level on admission (Group 1: ≥ 6 to < 7%, Group 2: ≥ 7 to < 8%, Group 3: ≥ 8 to < 9%, Group 4: ≥ 9 to < 10%, and Group 5: ≥ 10%). The frequency of hypoglycemia and factors associated with hypoglycemia were analyzed.

**Results:**

Hypoglycemia occurred in 15 patients (5.1%), including 4 (8%), 4 (6%), and 7 (10%) patients of Groups 1, 2, and 3, respectively, but in none of groups 4 and 5. Patients with hypoglycemia of Groups 1 had low insulin secretion and were high among insulin users, those of Groups 2 had low homeostasis model assessment of insulin resistance (HOMA-IR). Those of Group 2 and 3 had significantly lower mean blood glucose levels, those of Group 3 only had significantly lower maximum blood glucose level and percentage of AUC > 180 mg/dL. In any of the HbA1c groups, variations in blood glucose level were significantly larger in patients with hypoglycemia than without.

**Conclusions:**

Hypoglycemia occurred in patients with a wide range of HbA1c on admission (range 6–9%), suggesting that prediction of hypoglycemia based on HbA1c alone is inappropriate. Among patients with low HbA1c, strict control sometimes induce hypoglycemia. Among patients with high HbA1c, the possibility of hypoglycemia should be considered if there is a marked discrepancy between HbA1c and randomly measured blood glucose level. Larger variations in blood glucose level induce hypoglycemia in any of the HbA1c groups. The treatment to reduce variations in blood glucose level is important to prevent hypoglycemia.

## Background

The United Kingdom Prospective Diabetes Study and the ADVANCE Study demonstrated that strict blood glucose control can lessen the risk of microangiopathy [1, 2]. However, the Action to Control Cardiovascular Risk in Diabetes Study demonstrated increased overall death rate following strict blood glucose control, and sub-analysis of their data disclosed a higher annual death rate in the hypoglycemia episode group than in the hypoglycemia-free group [3, 4]. In recent years, numerous studies have demonstrated that repeated episodes of hypoglycemia increase the risk for cardiovascular disease and dementia and the death rate. Thus, it is important to avoid hypoglycemia during treatment of diabetes [5–8].

With regard to the clinical background of patients who develop hypoglycemia, it is thought that hypoglycemia tends to develop in patients with poorly controlled blood glucose, patients with low HbA1c, young patients, insulin users, and in elderly patients (contrary to the abovementioned report) and patients with long history of diabetes [8–10]. Another study reported that episodes of hypoglycemia are independent of HbA1c level [11]. To our knowledge, there is currently little or no information on the relation between hypoglycemia and HbA1c level or the factors associated with hypoglycemia.

In view of the known usefulness of continuous glucose monitoring (CGM) for evaluation of hypoglycemia among patients with type 2 diabetes mellitus (T2DM), the present study was designed to determine the association of frequency of hypoglycemia with HbA1c level and to identify the factors associated with hypoglycemia in hospitalized Japanese patients with T2DM, using CGM [12].

## Methods

### Subjects

The study subjects were inpatients with T2DM at the Hospital of The University of Occupational and Environmental Health, Japan for the purpose of education of diabetes and its affiliated hospitals between April 2010 and April 2015 with available blood glucose data recorded by CGM system (CGMS System Gold, Medtronic Inc., Fridley, MN; and iPro™2, Medtronic, Northridge, CA) within 5 days of admission while taking the same medications throughout the study period. The study population included patients of any age who were or were not taking glucose-lowering agents. In this study, we defined patients with T2DM as those with family history of diabetes and obesity, those without autoimmune diabetes, and those without hyperglycemia due to pancreatic failure or medication. The study excluded patients with type 1 diabetes, pancreatic diabetes, steroid diabetes, severe infection, pre- or postoperative status, and serious trauma. The study protocol was approved by the review board of The University of Occupational and Environmental Health, Japan and informed consent was obtained from all patients. The study conformed to the Declaration of Helsinki.

### Study protocol

In this retrospective study, 24-h CGM data were extracted from the second or third day of glucose monitoring. Patients had hospital meals at 25–30 kcal/kg standard body weight according to the dietary therapy recommended by the Japan Diabetes Society and underwent blood testing under fasting conditions on the second day of glucose monitoring [13]. For statistical analysis, the patients were stratified by HbA1c level on admission into five subgroups (≥ 6.0 to < 7.0%, ≥ 7.0 to < 8.0%, ≥ 8.0 to < 9.0%, ≥ 9.0 to < 10.0%, and ≥ 10%).

With regard to medications, information was collected about the number of oral glucose-lowering drugs used (percentages of patients using any such drug and patients using two or more such drugs) and about the use of insulin (percentages of insulin users and non-users).

The primary endpoint was the difference in the frequency of hypoglycemia among the different HbA1c groups. The secondary endpoint was the factors associated with episodes of hypoglycemia in each HbA1c subgroup.

### Biochemical and clinical measurements

The CGM devices used in this study included the Gold™ (Medtronic Inc.) and iPro™2 (Medtronic). The subcutaneous electrode measures glucose concentration in the interstitial fluid within a range of 40–400 mg/dL at a frequency of 288 times/day [14]. The sensor readings were calibrated against blood glucose levels measured in the morning, noon, and evening, and before bed (4 times/day). The glucose concentration measured by CGM is reported to correlate with the venous blood glucose level and is hereafter termed blood glucose level [14].

We excluded patients with microcirculatory impairment, which could potentially affect sensor performance. Data over 24 h extracted from the second or third day of glucose monitoring were used to calculate the average glucose level ± standard deviation (SD), mean amplitude of glycemic excursions (MAGE), coefficient of variation (CV), maximum blood glucose level, minimum blood glucose level, area under the blood concentration–time curve (AUC) > 180 mg/dL, percentage of AUC > 180 mg/dL, area over the blood concentration–time curve (AOC) < 70 mg/dL, and percentage of AOC < 70 mg/dL. MAGE was calculated using the Glycemic Variability Analyzer Program 1.1 (MATLABR 2010b) [15]. CV, Average glucose level′, SD′ and CV′ were calculated by the equation: CV = SD/average glucose level, Average glucose level′ = log_10_ (Average blood glucose level + 30), SD′ = log_10_ (SD + 30), CV′ = log_10_ (CV + 30) [16]. HbA1c (%) was determined using NGSP calculated by the equation: HbA1c (NGSP) (%) = HbA1c [Japan relationship of HbA1c (JDS) × 1.02 + 0.25 (%)] [17]. The equation used to calculate the estimated glomerular filtration rate (eGFR) was 194 × serum creatinine − 1.094 × age − 0.287 for men and 194 × serum creatinine − 1.094 × age − 0.287 × 0.739 for women. The homeostasis model assessment of insulin resistance (HOMA-IR) was calculated by the equation of fasting plasma glucose (mg/dL) × fasting plasma insulin (μU/mL)/405, and the urinary C-peptide reactivity (u-CPR) was determined in a 24-h pooled urine sample. HOMA-IR is not always correct in patients with blood glucose level > 180 mg/dL.

Patients were divided into five groups according to HbA1c level on admission (≥ 6 to < 7%, ≥ 7 to < 8%, ≥ 8 to < 9%, ≥ 9 to < 10%, and ≥ 10%), and data of each group were analyzed in terms of the presence/absence of hypoglycemia episodes. Hypoglycemia was defined as a CGM-based blood glucose level of < 70 mg/dL, regardless of the presence/absence of subjective symptoms. Blood glucose level < 50 mg/dL was considered severe hypoglycemia.

### Endpoints

The primary endpoint was the change in HbA1c levels at 24 weeks. The secondary endpoints were changes in urinary albumin excretion and LDL-C levels at 24 weeks.

### Statistical analysis

Data are expressed as mean ± SD. One-way analysis of variance (ANOVA) was used for comparison between groups, Wilcoxon was used for comparisons between the no hypoglycemia and hypoglycemia groups. The Chi square test was used to assess categorical data. Each test was performed at a significance level of 0.05. All statistical analyses were performed using JMP 11 (SAS Institute Inc., Cary, NC).

## Results

### Patient demographics

Table 1 shows the background variables of the study patients. The study included 293 patients (178 males and 115 females). Of these, 53, 64, 73, 49, and 54 patients were allocated to Group 1 (HbA1c (≥ 6 to < 7%), Group 2 (≥ 7 to < 8%), Group 3 (≥ 8 to < 9%), Group 4 (≥ 9 to < 10%), and Group 5 (≥ 10.0%), respectively. Patients of Group 5 were significantly younger with shorter duration of illness, and higher u-CPR. With regard to treatment, 51% of all patients of Group 1 were not using glucose-lowering drugs, whereas about half of the patients of Groups 2–4 were using dipeptidyl peptidase-4 (DPP-4) inhibitors. The percentage of insulin users was highest in Group 1 (25%) and lowest in Group 5 (2%).

**Table 1:** Clinical characteristics according to HbA1c level

	Group 1	Group 2	Group 3	Group 4	Group 5	P value*
n	53	64	73	49	54	
Age (years)	65.4 ± 1.7	65.9 ± 1.6	61.2 ± 1.5	63.3 ± 1.8	55.0 ± 1.8	< 0.001
Duration of diabetes (years)	12.1 ± 1.4	11.7 ± 1.3	10.4 ± 1.2	12.7 ± 1.5	6.2 ± 1.4	< 0.001
BMI (kg/m^2^)	25.2 ± 0.6	25.6 ± 0.6	26.5 ± 0.5	24.7 ± 0.6	26.9 ± 0.6	0.087
eGFR (mL/min/1.73 m^2^)	62.6 ± 3.6	66.1 ± 3.3	78.4 ± 3.0	74.7 ± 3.7	90.9 ± 3.5	< 0.001
HbA1c (%)	6.6 ± 0.1	7.4 ± 0.1	8.5 ± 0.1	9.5 ± 0.1	11.3 ± 0.1	< 0.001
FPG (mg/dL)	120.8 ± 5.0	134.4 ± 4.5	150.8 ± 4.2	164.2 ± 5.1	188.6 ± 4.9	< 0.001
HOMA-IR	2.4 ± 0.4 (n = 29)	2.5 ± 0.3 (n = 46)	2.9 ± 0.3 (n = 44)	2.2 ± 0.4 (n = 23)	2.5 ± 0.4 (n = 23)	0.628
Urinary CPR (μg/day)	66.0 ± 8.6	67.8 ± 7.4	81.5 ± 6.6	61.5 ± 8.2	107.0 ± 7.7	< 0.001
Treatment of diabetes						
Without glucose-lowering agents, n (%)	27 (51)	18 (28)	14 (19)	8 (16)	18 (33)	< 0.001
Sulfonylureas, n (%)	5 (9)	22 (34)	23 (32)	18 (37)	21 (39)	0.006
Biguanides, n (%)	4 (8)	16 (25)	16 (22)	15 (31)	11 (20)	0.058
DPP4i, n (%)	13 (25)	27 (42)	38 (52)	27 (55)	17 (31)	0.004
Thiazolidinedione, n (%)	6 (11)	8 (13)	7 (10)	6 (12)	7 (13)	0.978
Glinide, n (%)	1 (2)	0 (0)	0 (0)	2 (5)	0 (0)	0.154
αGI, n (%)	4 (8)	5 (8)	10 (14)	3 (6)	3 (6)	0.465
Insulin, n (%)	13 (25)	13 (20)	14 (19)	9 (18)	1 (2)	0.022
GLP-1, n (%)	1 (2)	1 (2)	2 (3)	1 (2)	3 (6)	0.699
Combination therapy						
Insulin only, n (%)	7 (13)	5 (8)	8 (11)	0 (0)	0 (0)	0.011
Without insulin						
Oral hypoglycemic drugs only 1, n (%)	8 (15)	15 (24)	18 (25)	14 (29)	17 (32)	0.336
Oral hypoglycemic drugs ≥ 2, n (%)	10 (19)	22 (35)	29 (40)	21 (43)	18 (34)	0.080
With insulin						
Oral hypoglycemic drugs only 1, n (%)	4 (8)	6 (9)	3 (4)	8 (16)	0 (0)	0.019
Oral hypoglycemic drugs ≥ 2, n (%)	2 (4)	2 (3)	3 (4)	1 (2)	1 (2)	0.939
All (n = 294)						
Hypoglycemia, n (%)	4 (8)	4 (6)	7 (10)	0 (0)	0 (0)	0.052
Severe hypoglycemia, n (%)	1 (2)	0 (0)	1 (1)	0 (0)	0 (0)	0.594
With glucose-lowering agents (n = 209) n	27	46	59	41	36	
Hypoglycemia, n (%)	4 (15)	4 (9)	7 (49)	0 (0)	0 (0)	0.044
Severe hypoglycemia, n (%)	1 (4)	0 (0)	1 (2)	0 (0)	0 (0)	0.451

Data are mean ± SD, unless otherwise indicated

*BMI* body mass index, *eGFR* estimated glomerular filtration rate, *HbA1c* hemoglobin A1c, *FPG* fasting plasma glucose, *HOMA-IR* homeostasis model assessment of insulin resistance, *CPR* C peptide immunoreactivity, *DPP4i* dipeptidyl peptidase-4 inhibitor, *αGI* α-glucosidase inhibitor, *GLP-1* glucagon-like peptide-1

* ANOVA for comparisons between each group, Chi square test for sex differences, treatment, hypoglycemia and severe hypoglycemia

### Hypoglycemia

**Fig. 1:**
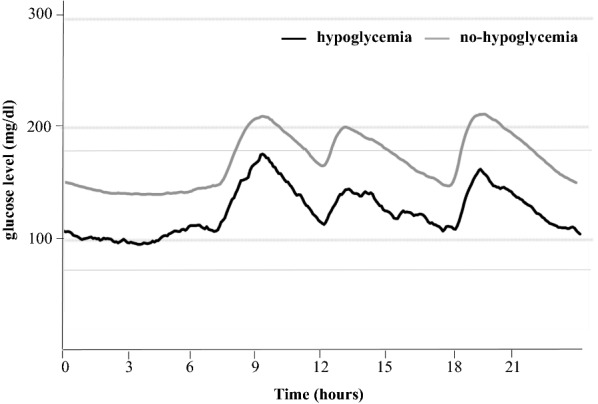
24-h glycemic variations ± 1SD in type 2 diabetes patients under treatment. Black line: hypoglycemia, gray line: without hypoglycemia. Continuous glucose monitoring (CGM) was applied for 2 or 3 days

Figure 1 shows 24-h glycemic variations ± 1SD with or without hypoglycemia. Table 1 shows the percentage of patients with hypoglycemia for each group. For the whole group, episodes of hypoglycemia were recorded in 15 (5.1%) patients; 4 patients (8%) of Group 1, 4 (6%) of Group 2, 7 (10%) of Group 3, and none of Groups 4 and 5. In other words, patients with HbA1c of ≥ 9% never developed hypoglycemia (p = 0.04). Severe hypoglycemia was seen in one patient each from Groups 1 and 3.

### Clinical characteristics of patients with hypoglycemia

**Fig. 2:**
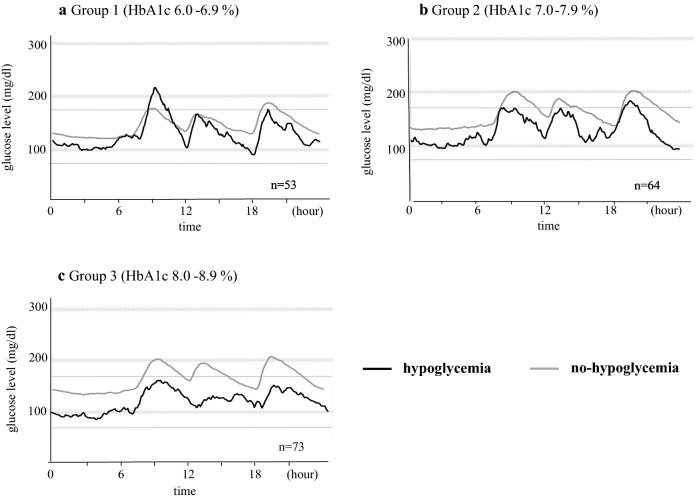
24-h glycemic variations ± 1SD in type 2 diabetes under treatment according to HbA1c levels. Black line: hypoglycemia, gray line: without hypoglycemia. **a** HbA1c 6.0–6.9%, **b** HbA1c 7.0–7.9%, **c** HbA1c 8.0–8.9%

Table 2 shows the clinical characteristics of patients stratified according to HbA1c level. Table 3 summarizes the clinical characteristics of patients of the different HbA1c groups, with and without hypoglycemia. Figure 2 shows 24-h glycemic variations ± 1SD in patients with or without hypoglycemia according to HbA1c level.

**Table 2:** Clinical characteristics of patients with or without hypoglycemia

	Group 1			Group 2			Group 3		
	No hypoglycemia	Hypoglycemia	P value*	No hypoglycemia	Hypoglycemia	P value*	No hypoglycemia	Hypoglycemia	P value*
N	49	4		60	4		66	7	
Age (years)	65.1 ± 1.8	69.5 ± 6.2	0.428	66.6 ± 1.6	54.5 ± 6.3	0.331	61.3 ± 1.7	60.1 ± 5.2	0.793
Duration of diabetes (years)	11.2 ± 1.9	22.3 ± 6.6	0.194	11.5 ± 1.5	14.8 ± 5.6	0.472	10.1 ± 1,1	13.4 ± 3.3	0.346
BMI (kg/m^2^)	25.4 ± 0.7	22.7 ± 2.4	0.239	25.6 ± 0.6	25.8 ± 2.5	0.824	26.5 ± 0.6	25.8 ± 1.8	0.415
eGFR (mL/min/1.73 m^2^)	62.9 ± 3.8	57.8 ± 13.2	0.711	66.5 ± 2.9	58.3 ± 12.8	0.571	79.5 ± 3.1	68.1 ± 9.5	0.382
HbA1c (%)	6.6 ± 0.0	6.6 ± 0.1	0.918	7.4 ± 0.1	7.5 ± 0.2	0.802	8.5 ± 0.0	8.4 ± 0.1	0.520
FPG (mg/dL)	120.2 ± 3.1	125.5 ± 10.7	0.410	135.3 ± 4.5	120.5 ± 17.4	0.533	152.8 ± 4.8	132.3 ± 14.7	0.166
HOMA-IR	2.4 ± 2.8 (n = 28)	0.7 ± 2.8 (n = 1)	0.555	2.7 ± 0.3 (n = 43)	0.8 ± 1.0 (n = 3)	0.020	2.6 ± 0.3 (n = 38)	4.7 ± 0.8 (n = 6)	0.505
Urinary CPR (μg/day)	68.8 ± 8.5	12.0 ± 37.6	0.037	70.6 ± 6.3	31.4 ± 22.5	0.080	84.1 ± 7.4	58.8 ± 21.8	0.115
Treatment of diabetes									
Without glucose-lowering agents, n (%)	26 (53)	0 (0)	0.041	18 (30)	0 (0)	0.196	14 (21)	0 (0)	0.076
Number of oral hypoglycemic agent, n	0.6 ± 0.1	0.5 ± 0.4	0.907	1.2 ± 0.2	1.5 ± 0.6	0.784	1.2 ± 0.1	1.9 ± 0.4	0.162
Sulfonylureas, n (%)	5 (10)	0 (0)	0.502	21 (35)	1 (25)	0.684	21 (32)	2 (29)	0.860
DPP4i, n (%)	11 (22)	2 (50)	0.218	25 (42)	2 (50)	0.744	32 (48)	6 (85)	0.060
Biguanides, n (%)	6 (12)	0 (0)	0.552	14 (23)	2 (50)	0.233	15 (23)	1 (14)	0.608
Thiazolidinedione, n (%)	6 (12)	0 (0)	0.457	7 (12)	1 (25)	0.435	6 (9)	1 (14)	0.657
Glinide, n (%)	1 (2)	0 (0)	0.758	0 (0)	0 (0)	–	0 (0)	0 (0)	–
αGI, n (%)	4 (8)	0 (0)	0.552	5 (8)	0 (0)	0.548	8 (12)	2 (29)	0.229
Insulin, n (%)	10 (20)	3 (75)	0.015	10 (17)	1 (25)	0.669	13 (20)	1 (14)	0.730
GLP-1, n (%)	1 (2)	0 (0)	0.766	1 (2)	0 (0)	0.819	2 (3)	0 (0)	0.632
Combination therapy									
Insulin only, n (%)	5 (10)	2 (50)	0.024	4 (7)	1 (25)	0.186	8 (12)	0 (0)	0.329
Without insulin									
Oral hypoglycemic drugs only 1, n (%)	6 (12)	2 (50)	0.042	13 (22)	2 (50)	0.213	15 (23)	3 (43)	0.251
Oral hypoglycemic drugs ≥ 2, n (%)	10 (20)	0 (0)	0.316	21 (36)	1 (25)	0.651	26 (40)	3 (43)	0.884
With insulin									
Oral hypoglycemic drugs only 1, n (%)	3 (6)	1 (25)	0.169	5 (8)	0 (0)	0.268	2 (3)	1 (14)	0.154
Oral hypoglycemic drugs ≥ 2, n (%)	2 (4)	0 (0)	0.680	2 (3)	0 (0)	0.711	3 (5)	0 (0)	0.565
Glycemic variations									
Average glucose level 0–24 h (mg/dL)	146.0 ± 4.6	127.2 ± 16.0	0.232	158.8 ± 3.3	125.7 ± 12.6	0.023	169.0 ± 3.7	120.1 ± 11.2	< 0.001
SD 0–24 h (mg/dL)	30.9 ± 7.0	30.9 ± 2.0	0.225	33.7 ± 1.9	34.5 ± 7.2	0.598	36.6 ± 1.5	35.3 ± 4.6	0.844
MAGE 0–24 h (mg/dL)	88.2 ± 3.5	110.5 ± 12.3	0.381	96.5 ± 4.2	109.3 ± 16.2	0.305	98.5 ± 3.1	94.8 ± 9.5	0.555
Maximum glucose level 0–24 h (mg/dL)	223.3 ± 8.0	223.3 ± 8.0	0.893	237.6 ± 6.9	213.5 ± 26.8	0.332	256.3 ± 5.6	194.7 ± 17.1	< 0.001
Minimum glucose level 0–24 h (mg/dL)	98.6 ± 2.8	56.3 ± 10.0	< 0.001	105.1 ± 2.8	62.0 ± 10.8	< 0.001	108.1 ± 3.2	60.0 ± 9.9	< 0.001
CV (%)	0.21 ± 0.01	0.30 ± 0.03	0.031	0.21 ± 0.01	0.27 ± 0.04	0.133	0.22 ± 0.01	0.29 ± 0.02	< 0.001
Average glucose level′ 0–24 h (mg/dL)	2.23 ± 0.01	2.18 ± 0.03	0.114	2.26 ± 0.01	2.22 ± 0.03	0.148	2.28 ± 0.01	2.16 ± 0.03	< 0.001
SD′ 0–24 h (mg/dL)	0.07 ± 0.00	0.11 ± 0.01	0.020	0.08 ± 0.00	0.10 ± 0.01	0.010	0.08 ± 0.00	0.10 ± 0.01	0.071
CV′ (%)	3.18 ± 0.13	4.81 ± 0.44	0.011	3.35 ± 0.16	4.60 ± 0.65	0.068	3.52 ± 0.15	4.59 ± 0.45	0.027
Percentage of AUC > 180 0–24 h (%)	17.6 ± 10.1	12.6 ± 10.1	0.735	26.7 ± 2.7	8.8 ± 10.4	0.076	34.5 ± 2.9	7.3 ± 8.9	< 0.001
Percentage of AOC < 70 0–24 h (%)	0	4.5 ± 0.4	< 0.001	0	4.2 ± 0.3	< 0.001	0	8.1 ± 1.3	< 0.001

Data are mean ± SD, unless otherwise indicated

*SD* standard deviation, *MAGE* mean amplitude of glycemic excursions, *CV* coefficient of variation, Average glucose level′= log_10_ (Average glucose level +30); SD′ = log_10_ (SD + 30); CV′ = log_10_ (CV + 30); *AUC* area under the blood concentration–time curve, *AOC* area over the blood concentration–time curve. See Table 1 for abbreviations

* Wilcoxon for comparisons between the no hypoglycemia and hypoglycemia groups, Chi square test for sex differences

**Table 3:** Characteristics of individual patients with hypoglycemia

	Sex/age	BMI (kg/m^2^)	DM duration (years)	Blood glucose level (mg/dL)	HbA1c (%)	HOMA-IR	Urinary CPR (μg/day)	Therapy
1	M/72	23.8	1	64	6.4	–	–	DPP4i
2	F/75	20.8	25	63	6.4	0.7	12.9	Insulin mix50
3	M/58	21.2	38	56	6.8	–	–	Insulin, DPP4i
4	F/73	25.2	25	42	6.9	–	11.1	Insulin mix30
5	F/17	21.0	5	65	7.3	0.8	29.3	Biguanides
6	F/57	30.9	5	60	7.4	1.1	75.6	DPP4i
7	M/74	23.7	17	64	7.6	–	1.1	Insulin mix25
8	M/70	27.4	32	62	7.6	0.7	19.4	Sulfonylureas, DPP4i, biguanides, Thiazolidinedione
9	F/79	15.9	13	67	8	–	11.6	Insulin, αGI
10	F/67	22.0	9	65	8.1	5.9	41.7	DPP4i, glinide, αGI
11	M/34	27.3	4	57	8.4	0.9	14.4	DPP4i
12	M/70	34.0	11	65	8.5	9.6	104.8	DPP4i
13	F/70	22.4	25	47	8.5	0.9	32	Sulfonylureas, DPP4i, Thiazolidinedione
14	M/36	38.4	2	59	8.6	9.0	182.1	DPP4i
15	F/65	20.6	30	60	8.7	1.8	24.9	Sulfonylureas, DPP4i, biguanides

See Table 1 for abbreviations

For patients of Group 1, **t**he u-CPR was significantly lower in the hypoglycemia group (12.0 μg/day, n = 5) than those free of hypoglycemia (68.8 μg/day, n = 49). Patients with hypoglycemia of Groups 1 were high among insulin users (5.1%, p = 0.015). The hypoglycemia group included not only insulin users but also users of DPP-4 inhibitor. Of the insulin users of the hypoglycemia group, 2 patients used an insulin mixture and 1 patient was on intensive insulin therapy combined with DPP-4 inhibitor therapy. One of the two users of insulin mixture developed severe hypoglycemia. Moreover, one patient developed hypoglycemia during treatment with a DPP-4 inhibitor alone.

With regard to patients of Group 2, HOMA-IR was lower in the hypoglycemia group than hypoglycemia-free group. Diverse drugs were being used by patients of the hypoglycemia subgroup (DPP-4 inhibitor by 1 patient, biguanide alone by 1, multiple oral glucose-lowering drugs by 1, and insulin mixture by 1 patient), but none developed severe hypoglycemia.

Patients with hypoglycemia of Groups 3 had significantly higher HOMA-IR. For medications used in the hypoglycemia group, 6 of the 7 patients used DPP-4 inhibitors, and half of these 6 patients also used insulin secretion stimulators [e.g., sulfonylureas (SU) and glinide] in combination with DPP-4 inhibitors. One of these patients developed severe hypoglycemia. This patient received three-drug combination therapy with DPP-4 inhibitor, high-dose SU, and thiazolidinedione. Hypoglycemia also occurred in a patient who received intensive insulin therapy plus an α-glucosidase inhibitor.

### CGM parameters of patients with hypoglycemia

For Groups 1, the mean blood glucose level was not different between those with and without hypoglycemia. For Group 2 and 3, the mean blood glucose level was significantly lower in patients with hypoglycemia (120.1 mg/dL) than without (169.0 mg/dL). Furthermore, the maximum glucose level and proportion of AUC > 180 mg/dL were significantly lower in patients who developed hypoglycemia than those free of hypoglycemia in Group 3 only. In Groups 1–3, the minimum glucose level and proportion of AOC < 70 mg/dL were significantly lower for patients with hypoglycemia than without. With regards to the markers of changes in blood glucose level, CV, SD′ and CV′ in Groups 1, SD′ in Groups 2 and CV, CV′ in Groups 3 were significantly larger in patients with hypoglycemia than without.

## Discussion

In the present study, hospitalized patients with type 2 diabetes mellitus were evaluated to determine the status of hypoglycemia in relation to their HbA1c level, using CGM. Episodes of hypoglycemia were noted in patients with a wide range of HbA1c levels (6–9%), corroborating the previously reported finding that prediction of hypoglycemia is not possible on the basis of HbA1c alone [18]. Noteworthy, although some investigators reported that hypoglycemia often develops in poorly controlled diabetic patients [8], hypoglycemia in the present study was not recorded in any of the patients of the poorly controlled groups (HbA1c ≥ 9%). This finding is probably related to the following factors: (1) the poorly controlled patients (high HbA1c groups) in the present study were relatively young; (2) the duration of diabetes in these patients was short; (3) the percentage of insulin users was low; (4) 33% of all patients were under diet therapy alone; and (5) the percentage of patients recently diagnosed with diabetes and with retained insulin secreting capability was high.

The overall incidence of hypoglycemia in this study was 5.1%, which is much lower than that of 49.1% reported in a previous study using CGM [12]. It has been suggested that the risk for hypoglycemia increases in patients with T2DM on combination treatment with both insulin and oral glucose-lowering drugs [19]. The percentage of patients using insulin secretion stimulators (e.g., SU, glinide) was approximately 30% in both the previous study and in the present study, whereas the percentage of insulin users was lower in the present study (17%) than in the previous study (70%). This difference may be the cause of the different incidence of hypoglycemia between the present and previous study.

With regard to clinical features of diabetic patients who developed hypoglycemia, patients of Group 1 had significantly lower insulin secretion and were high among insulin users in the present study. If this result is combined with the previous report that the risk for hypoglycemia is higher in insulin users, it seems that strict blood glucose control by insulin therapy increases the risk of hypoglycemia [19]. Some papers have reported localized amyloidosis at the site of repeated insulin injection in a diabetic patient was the risk for hypoglycemia, it might be a reason of hypoglycemia in insulin users [20].

Furthermore, insulin users who developed hypoglycemia were often using an insulin mixture, and one of these patients developed severe hypoglycemia. A previous study that compared patients on insulin mixture therapy with those on basal-bolus therapy demonstrated significantly high incidence of hypoglycemia in the insulin mixture therapy group [21]. The results of the present study do not contradict the findings of this previous study [21].

On the other hand, poorly controlled patients who developed hypoglycemia in Group 3 had significantly higher HOMAIR. Thirty % of the patients who developed hypoglycemia were receiving DPP-4 inhibitor + SU therapy. Although DPP-4 inhibitors are considered safe drugs, and unlikely to cause hypoglycemia, it has been reported that their use in combination with SU enhances the drug activity, mediated by the Epac2A/Rap1 signaling pathway, consequently leading to 50% rise in the risk of hypoglycemia [22, 23]. In addition, cases of noninsulinoma pancreatogenous hypoglycemia due to excessive insulin secretion, regardless of the presence/absence of underlying diabetes mellitus, have been reported, at least partly explaining the episode of hypoglycemia in patients receiving DPP-4 inhibitors alone [24].

In the analysis of CGM data of Groups 1–3, the mean blood glucose and were significantly lower for the hypoglycemia patients than hypoglycemia-free patients in Group 1 and 2, maximum glucose levels, as well as the proportion of AUC > 180, were significantly lower for the hypoglycemia patients than hypoglycemia-free patients only in Group 3, whereas the proportion of AOC < 70 was significantly higher in the hypoglycemia group in all three HbA1c groups. These results highlight the importance of ongoing hypoglycemia and that it should be considered in patients showing discrepancies between HbA1c and randomly measured blood glucose levels even when the HbA1c level is high. In addition, one previous report stated that the rate of hypoglycemia among insulin users tended to increase along with increasing variations in blood glucose level [25]. In the present study, the variations in blood glucose level were significantly larger for the hypoglycemia patients than hypoglycemia-free patients in any of the HbA1c groups. In Group 1, the percentage of insulin users was high among the hypoglycemia cases. The treatment to reduce variations in blood glucose level is important to prevent hypoglycemia.

As mentioned above, the results of CGM suggest that prediction of hypoglycemia is not possible with HbA1c alone. Furthermore, in the low HbA1c groups, insulin secretion was lower and the incidence of hypoglycemia were high among insulin users, suggesting that strict blood glucose control can induce hypoglycemia. In the high HbA1c groups, it seems necessary to consider the possibility of hypoglycemia if a discrepancy between HbA1c and randomly measured blood glucose levels is noted.

The present study has several limitations, including (1) its retrospective study design, (2) small sample size, (3) evaluation of hospitalized patients with discrepancies between HbA1c on admission and blood glucose level after admission, (4) the fact that the CGM data were lower than the actual self-monitored blood glucose levels (possible exaggeration of this difference during hypoglycemia), and (5) high percentage of patients receiving multiple drugs, possibly modifying hypoglycemia. Further prospective studies of large number of outpatients, including larger number of patients receiving no medication, are needed to confirm the present findings.

## Abbreviations

T2DM: type 2 diabetes mellitus
HbA1c: glycated hemoglobin
CGM: continuous glucose monitoring
HOMA-IR: homeostasis model assessment of insulin resistance
SD: standard deviation
MAGE: mean amplitude of glycemic excursion
CV: coefficient of variation
AUC: area under the blood concentration–time curve
AOC: area over the blood concentration–time curve
eGFR: estimated glomerular filtration rate
uCPR: urinary C-peptide reactivity
SU: sulfonylureas
